# Platelets can be a biological compartment for the Hepatitis C Virus

**DOI:** 10.1590/S1517-838246220140553

**Published:** 2015-06-01

**Authors:** Jovita Ramos Ariede, Maria Inês de Moura Campos Pardini, Giovanni Faria Silva, Rejane Maria Tommasini Grotto

**Affiliations:** 1 Universidade Estadual Paulista, Divisão Hemocentro, Faculdade de Medicina de Botucatu, Universidade Estadual Paulista, Botucatu, SP, Brasil, Programa de Pós-Graduação em Pesquisa e Desenvolvimento (Biotecnologia Médica), Divisão Hemocentro, Faculdade de Medicina de Botucatu, Universidade Estadual Paulista "Júlio de Mesquita Filho", Botucatu, SP, Brasil.; 2 Universidade Estadual Paulista, Departamento de Clínica Médica, Faculdade de Medicina de Botucatu, Universidade Estadual Paulista, Botucatu, SP, Brasil, Departamento de Clínica Médica, Faculdade de Medicina de Botucatu, Universidade Estadual Paulista "Júlio de Mesquita Filho", Botucatu, SP, Brasil.; 3 Universidade Estadual Paulista, Divisão Hemocentro, Faculdade de Medicina de Botucatu, Universidade Estadual Paulista, Botucatu, SP, Brasil, Laboratório de Biologia Molecular, Divisão Hemocentro, Faculdade de Medicina de Botucatu, Universidade Estadual Paulista "Júlio de Mesquita Filho", Botucatu, SP, Brasil.; 4 Universidade Estadual Paulista, Faculdade de Ciências Agronômicas, Fazenda Experimental de Lageado, Universidade Estadual Paulista, Botucatu, SP, Brasil, Departamento Bioprocessos e Biotecnologia, Faculdade de Ciências Agronômicas, Fazenda Experimental de Lageado, Universidade Estadual Paulista "Júlio de Mesquita Filho", Botucatu, SP, Brasil.

**Keywords:** Hepatitis C Virus, platelets, viral load, virological relapse

## Abstract

Although HCV has hepatic tropism, the presence of the virus in extra-hepatic compartments has been well documented. Platelets have been described as carriers of the virus in the circulation and may be a natural reservoir for the virus. However, few studies have been performed to evaluate the levels of HCV RNA in plasma and platelets are equal or differ in some way. Therefore, the aim of this study was to perform a comparative evaluation of the stability of HCV RNA in plasma and isolated platelets. Four aliquots of whole plasma obtained from patients infected with HCV were incubated at 37 °C for 0, 48, 96 and 144 h. After incubation, the plasma and platelet pellet was obtained from each aliquot. Viral RNA in plasma and platelets was quantified by q-PCR. The results showed a decrease in HCV RNA levels in plasma with incubation time. However, platelet HCV RNA levels were stable up to 144 h incubation. The results of this study showed that HCV RNA in platelets, although at lower concentrations than in plasma, is preserved from degradation over time, suggesting that the virus may persist longer in the body when associated with platelets, which could have an impact on the efficiency of antiviral therapy.

## Introduction

The study of the viral kinetics of infection by the hepatitis C virus (HCV) still presents difficulties due to lack of experimental models (Penin *et al.*, 2004). HCV replication models show that adaptive mutations frequently occur regardless of virus variants and can promote virus replication (Bartosh and Cosset, 2006). Thus, the pathogenesis of HCV infection differs from classical models of infection by viruses, reflecting the adaptation to humans (Alter and Houghton, 2001).

In the course of chronic infection by the virus, phases of high-level viremia may alternate with phases of low-level viremia or undetectable virus in plasma or serum (Espírito-Santo *et al.*, 2013). However, studies have demonstrated that HCV is not only found on hepatocytes, but also plasma and/or serum of infected patients (Fujiwara *et al.*, 2013). Although the hepatocyte is the major target cell of HCV, viral RNA has been found in extrahepatic compartments (Schmidt *et al.*, 1997; Castillo *et al.*, 2005; Chary *et al.*, 2012). Furthermore, studies have demonstrated that in individuals infected with HCV, viral RNA is associated with platelets, which are carriers of the virus in circulation (Hamaia *et al.*, 2001; De Almeida *et al.*, 2009; Padovani *et al.*, 2013).

Few studies have been performed to evaluate the amount of viral RNA in other biological compartments, so it is not yet well established whether the quantification of HCV RNA in plasma or serum differs from that found in other biological compartments (Hamaia *et al.*, 2001; Chary *et al.*, 2012; Espírito-Santo *et al.*, 2013).

Therefore, the aim of this study was to perform a comparative evaluation of the stability of HCV RNA in plasma and isolated platelets.

## Materials and Methods

Aliquots of ethylenediaminetetraacetic acid-anticoagulated peripheral venous blood were collected from HCV-infected patients who were antiviral treatment-naïve and that would initiate antiviral treatment during the development of the study and had HBV- and HIV-negative serology and the absence of other hepatic diseases, seen at the Department of Internal Medicine, Gastroenterology Division, Botucatu Medical School, Sao Paulo State University, UNESP, Botucatu, SP, Brazil.

The study was approved by the Research Ethics Committee of Botucatu Medical School, UNESP (number 3764-2011).

The blood sample was centrifuged for 3 min at 1312 g to obtain platelet-rich plasma, which was separated into four aliquots. The first one was centrifuged for 5 min at 1600 g to pellet the platelets. The supernatant was removed, and the platelet pellet was washed with 0.9% NaCl five times.

The same procedure was performed with the other three aliquots, after incubation for 48, 96 and 144 h, respectively, at 37 °C in an Incubator Shaker (New Brunswick Scientific, USA) with horizontal mixing at 30 g until RNA quantification.

HCV RNA quantification in plasma and platelets was performed according Padovani *et al.* (2013).

HCV genotyping was performed in plasma and platelet samples using 5′ UTR and core genomic region according to Levada *et al.* (2010).

This experiment was performed in triplicate. Two negative controls were used in the experiment according to all Padovani *et al.* (2013). The first was performed with platelet pellet and these were incubated with the HCV-negative serum, and the second consisted in an empty tube without platelets, and this tube was incubated with the same set of serum used in test samples.

After the last washed, the supernatant was used as source to RT-PCR for HCV identification according Levada *et al.* (2010) for evaluate any possibility of contamination with serum HCV.

## Results

The samples were obtained from patients with a mean age of 57.5 years [IQR (35.5–87.5)], two men and a woman. All patients presented with portal fibrosis in F2/F3 according to the METAVIR score.

Table 1 summarizes the HCV RNA levels in plasma and platelets for three replicates performed according to the processing time: 0 (immediately after collection), and after 48, 96, 144 h of incubation at 37 °C.

**Table 1:** HCV RNA Levels in plasma and platelets according to processing time: 0 h (immediately after collection) and after 48, 96, 144 h of incubation at 37 °C.

Plasma	0 h (Log)	48 h (Log)	96 h (Log)	144 h (Log)
Replicate 1	6.08	4.74	3.61	2.82
Replicate 2	7.75	7.64	3.55	3.39
Replicate 3	7.76	7.22	4.54	2.31

Platelets	0 h (Log)	48 h (Log)	96 h (Log)	144 h (Log)

Replicate 1	3.13	3.20	3.41	3.60
Replicate 2	4.94	5.82	5.88	5.92
Replicate 3	4.65	5.50	5.77	5.87

The results showed a decrease in plasma HCV RNA levels with incubation time. However, platelet HCV RNA levels were stable up to 144 h incubation.

The HCV genotype present in plasma and platelets was concordant (genotype 3a).

No contaminations were detected in the experiments.

## Discussion

The results of our study demonstrate that HCV RNA in platelets, although at a lower concentration than in plasma, is more preserved from degradation over time (Table 1), suggesting that the virus may persist longer in the body when associated with platelets.

These findings suggest the presence of HCV in platelets as a biological compartment of reservation. Hamaia *et al.* (2001) have suggested that, besides the possibility of specific surface molecules mediating HCV binding to platelets, the morphology of these cells could result in the nonspecific adsorption of HCV on the platelet surface. Based on this hypothesis, probably higher viral load values could be associated with the detection of HCV in platelets.

The nature of HCV binding to cells is not well understood and a number of potential receptors have been investigated (Bartosh, 2006). The hypervariable region 1 of HCV E2 envelope glycoproteins appears to play a central role in the HCV binding to cells, although CD81 has been postulated as a major HCV receptor (De Almeida *et al.*, 2007; Marincola and Alter, 2013).

In this line of reasoning, the platelets can function as an important compartment for the virus and sequester the virions from the circulation, contributing to viral persistence and viral escape from the host immune system, which may have an impact on the efficiency of antiviral therapy (Alter and Houghton, 2001).

Further studies with an increase number of samples in this population are necessary to better evaluate the dynamics of HCV RNA preservation of platelets over time.

The results of this study open perspectives for viruses associated with platelets to be the target of therapeutic intervention strategies, since this type of virus is more efficiently sequestered in this biological compartment than in plasma.

## References

[B01] Alter JJ, Houghton M (2001). Clinical Medical Research Award Hepatitis C virus and eliminating
post-transfusion hepatitis. Nat Med.

[B02] Bartosh B, Cosset FL (2006). Cell entry of hepatitis C virus. Virology.

[B03] Castillo I, Rodríguez-Iñigo E, Bartolomé J (2005). Hepatitis C virus replicates in peripheral blood mononuclear
cells of patients with occult hepatitis C virus infection. Gut.

[B04] Chary A, Winters MA, Eisen R (2012). Quantitation of hepatitis C virus RNA in peripheral blood
mononuclear cells in HCV-monoinfection and
HIV/HCV-coinfection. J Med Virol.

[B05] De Almeida AJ, Campos-de-Magalhães M, Brandão-Mello CE (2007). Detection of hepatitis C virus in platelets: evaluating its
relationship to viral and host factors. Hepatogastroenterology.

[B06] De Almeida AJ, Campos-De-Magalhães M, Brandão-Mello CE (2009). Detection of hepatitis C virus in platelets: evaluating its
relationship to antiviral therapy outcome. Hepatogastroenterology.

[B07] Espírito-Santo MP, Brandão-Mello CE, Marques VA (2013). Analysis of hepatitis C virus (HCV) RNA load in platelets of
HCV-monoinfected patients receiving antiviral therapy. Ann Hepatol.

[B08] Fujiwara K, Allison RD, Wang RY (2013). Investigation of residual hepatitis C virus in presumed recovered
subjects. Hepatology.

[B09] Hamaia S, Li C, Allain JP (2001). The dynamics of hepatitis C virus binding to platelets and 2
mononuclear cell lines. Blood.

[B10] Levada PM, Moraes CF, Corvino SM (2010). Reverse hybridization and sequencing for genotyping the hepatitis
C virus. Rev Soc Bras Med Trop.

[B11] Padovani JL, Corvino SM, Drexler JF (2013). In vitro detection of hepatitis C virus in platelets from
uninfected individuals exposed to the virus. Rev Soc Bras Med Trop.

[B12] Penin F, Dubuisson J, Rey FA (2004). Structural biology of hepatitis C virus. Hepatology.

[B13] Rehermann B (2013). Pathogenesis of chronic viral hepatitis: differential roles of T
cells and NK cells. Nature Medicine.

[B14] Schmidt WN, Wu P, Han JQ (1997). Distribution of Hepatitis C Virus (HCV) RNA in whole blood and
blood cell fractions: plasma HCV RNA analysis underestimates circulating
virus load. J Infec Dis.

